# OLDER ADULTS’ IMMIGRANT STATUS AND SELF-REPORTED ABILITY TO NAVIGATE THROUGH THE HEALTHCARE SYSTEM

**DOI:** 10.1093/geroni/igz038.1838

**Published:** 2019-11-08

**Authors:** Huey-Ming Tzeng

**Affiliations:** University of Saskatchewan, Saskatoon, Saskatchewan, Canada

## Abstract

This exploratory study examined the association between older adults’ immigrant status and their self-reported ability to perform each of the 51 self-care behaviors that are needed for them to navigate through the healthcare system. Secondary data analysis was conducted based on a 2018 telephone survey of community-dwelling adults 65 y/o or older, living in a western Canada province (N = 1,000). A previously validated survey tool, Patient Involvement Behaviors in Health Care (e.g., indicating Yes=1 or No=0 regarding their ability to perform each self-care behavior), and a demographic data form (e.g., are you an immigrant? Yes=1 or No=0) were used. Descriptive analyses and chi-square tests for independence (alpha= 0.05) were conducted. Among the 993 adults who indicated their immigrant status, 51 (5.1%) self-declared as immigrants. 32 (62.7%) of the immigrant participants and 457 (48.5%) of the non-immigrant participants resided in the urban areas. 88.2% of these immigrant participants was white, 7.8% was Asian, and 2% was black; 72.5% indicated that English is their first language. Immigrant participants were less likely to report being able to perform 5 self-care behaviors than non-immigrant participants. These 5 behaviors were: bringing someone to help you move around when needed; asking your providers to share your medical record with each other; finding insurance that best matches your needs; changing health insurance coverage as needed; and knowing of any interactions with old and new treatments. Clinicians should co-create approaches with older adult immigrants to improve their self-care capacity (e.g., connecting with relevant peer support networks).

